# Inhibitory Activity of Natural Synergetic Antimicrobial Consortia Against *Salmonella enterica* on Broiler Chicken Carcasses

**DOI:** 10.3389/fmicb.2021.656956

**Published:** 2021-04-29

**Authors:** Liya Zhang, Laila Ben Said, Moussa Sory Diarra, Ismail Fliss

**Affiliations:** ^1^Institute of Nutrition and Functional Foods, Université Laval, Québec, QC, Canada; ^2^Guelph Research and Development Centre, Agriculture and Agri-Food Canada, Guelph, ON, Canada

**Keywords:** chicken carcass, natural antimicrobials, microcin J25, reuterin, lactic acid, *Salmonella*

## Abstract

The currently most utilized antimicrobial agent in poultry processing facilities is peracetic acid, a chemical increasingly recognized as hazardous to human health. We evaluated the efficacy of mixtures of natural antimicrobial compounds, namely reuterin, microcin J25, and lactic acid, for reducing the viability of *Salmonella enterica* and total aerobes on broiler chicken carcasses. The compounds were compared singly and in combination with water and 0.1% peracetic acid. The minimum inhibitory concentrations of reuterin, lactic acid, and microcin J25 against *S. enterica* serovar Enteritidis were respectively 2 mM, 0.31%, and 0.03 μM. *In vitro*, the combinations of reuterin + lactic acid and reuterin + microcin J25 were synergic, making these compounds effective at four times lower concentrations than those used alone. *Salmonella* viable counts fell to zero within 10 min of contact with reuterin + lactic acid at 10 times the concentrations used in combination, compared to 18 h in the case of reuterin + microcin J25. Sprayed onto chilled chicken carcasses, this reuterin + lactic acid mixture reduced *Salmonella* spp. counts by 2.02 Log CFU/g, whereas reuterin + microcin J25 and peracetic acid reduced them by respectively 0.83 and 1.13 Log CFU/g. The synergy of reuterin with lactic acid or microcin J25 as inhibitors of bacterial growth was significant. Applied as post-chill spray, these mixtures could contribute to food safety by decreasing *Salmonella* counts on chicken carcasses.

## Introduction

Nontyphoidal serovars of *Salmonella enterica*, commonly associated with poultry, pose a well-known health risk. As foodborne pathogens, they cause much hospitalization, sometimes with fatal outcome. About 35% of foodborne illnesses traceable to poultry are due to *S. enterica* at a social cost of about $700 million annually (Morris et al., 2011). The prevalence of *Salmonella* on processed poultry meat has been estimated at 20–43% (Scheinberg et al., 2013; Trimble et al., 2013). According to the Canadian Food Inspection Agency (2018), all the holders of licenses to produce poultry for distribution are expected to have control strategies in place to eliminate microbial pathogens or prevent them from reaching dangerous levels. The Performance Standard for *Salmonella* in young chicken carcasses tolerates up to five positive test results in a set of 51 samples (Annex U: USDA, 2017). Poultry producers and processors must therefore employ efficient preventive strategies of *Salmonella* control throughout the chain.

A variety of treatments have been reported to reduce the microbial load of chicken carcasses after slaughter. These include hot water, infrared radiation, gamma radiation, and spray/chill systems enhanced with chemicals such as chlorine, trisodium phosphate, hydrogen peroxide, ozonated water, and ethanol (El-Ziney et al., 1999). Furthermore, a wide range of organic acids have been considered and tested in poultry products. The lack of safety and the chemical nature of most of these compounds make their use in the food sector controversial. The increasing demand of the consumers for safer and natural compounds makes the development of new alternatives urgently needed. Previously, chlorine has been utilized as one of the primary antimicrobial agents in poultry processing plants for carcass decontamination; but replaced with peracetic acid during the past decade. Obtained by combining acetic acid and hydrogen peroxide, peracetic acid is effective at concentrations of 200–2000 ppm in aqueous solution. However, it is unstable, corrosive, and reportedly an irritant to the upper respiratory tract, eyes, and skin (Dittoe et al., 2019). When used for decontamination purposes, it can cause undesirable color, texture, and flavors to develop in chicken products. The search continues for the means of ensuring food safety while maintaining quality using milder alternative natural compounds.

Bio-preservation refers to the use of microorganisms and/or their metabolites to increase product shelf life and ensure food safety. Lactic acid bacteria are able to produce a variety of antimicrobial substances, including organic acids, bacteriocins, and low-molecular-mass compounds such as short-chain fatty acids and reuterin (Reis et al., 2012; Hernández-Aquino et al., 2019). Reuterin (3-hydroxypropionaldehyde) is a neutral broad-spectrum antimicrobial compound produced from glycerol by *Lactobacillus reuteri*. It is water-soluble, effective over a wide pH range and not inactivated by enzymes (El-Ziney et al., 1999). These advantages make it suitable as a preservative in a variety of foods including meat and poultry products. Bacteriocins are proteinaceous molecules exhibiting bacteriostatic or bactericidal activities covering relatively narrow spectra of bacterial taxa, generally related closely to the producing strain. They act by forming pores in cell membranes and/or inhibiting cell wall synthesis (Roces et al., 2012). Produced by *Escherichia coli*, microcin J25 is bactericidal to several Gram-negative foodborne pathogens including *E. coli* and *Salmonella*. Its peculiar lasso structure makes it highly resistant to thermal denaturation. Though attractive to the food industry, it is being adopted very slowly in large part because of its narrow spectrum of activity, sensitivity to food enzymes, and the possible development of resistant variants of pathogens.

In general, bacteriocins produced by Gram-positive bacteria have little or no impact on the viability of Gram-negative bacteria such as *Salmonella*. This resistance is due to the outer membrane, which acts as a barrier against the diffusion of large molecules such as proteins and hydrophobic substances including some antibiotics (Prudencio et al., 2016). The use of synergic combinations of compounds represents a promising strategy to overcome this obstacle. Counts of *S. enterica* in soybean sprouts have been reduced significantly using washing solutions containing enterocin AS-48 with lactic, polyphosphoric, peracetic or hydrocinnamic acids, or sodium hypochlorite (Molinos et al., 2008). Inhibition of planktonic and biofilm cultures of *E. coli* by colistin has been enhanced with nisin/enterocin (Al Atya et al., 2016). Bovicin HC5 appears to be effective against *Salmonella* when combined with EDTA (Prudencio et al., 2016). Nisin and high hydrostatic pressure appear to enhance each other as inactivators of total aerobic bacteria (Zhao et al., 2013). The use of antimicrobial combinations also makes the compounds effective at lower concentrations and the emergence of resistant variants much less likely (Bassetti and Righi, 2015; Gupta and Datta, 2019).

The objective of the present study was to evaluate reuterin, microcin J25, and lactic acid separately and in combination as natural-sourced inhibitors of *Salmonella* on broiler chicken carcasses.

## Materials and Methods

### Bacterial Strains and Culture Media

*Salmonella enterica* serovar Enteritidis MNHN kindly provided by Prof. Sylvie Rebuffat (Muséum national d’Histoire naturelle, Paris, France), *Salmonella* Heidelberg CMBL4-8 (Université Laval METABIOLAC collection), and *Salmonella* Newport ATCC 6962 were used as test strains for antibacterial activity assays. *Lactobacillus reuteri* from broiler chicken intestine (isolate C1-14, unpublished) and *E. coli* MC4100 carrying the pTUC202 plasmid were used respectively for reuterin and microcin J25 production. *Salmonella* strains were maintained as glycerol stock at −80°C and cultured in nutrient broth (NB, Oxoid) at 37°C for 18 h prior to use. *Lactobacillus reuteri* was maintained in MRS broth (Nutri Bact, Terrebonne, Canada) and cultured at 37°C for 18 h under anaerobic conditions (Forma Scientific, United States). *Escherichia coli* was cultured at 37°C overnight under aerobic conditions in Luria-Bertani (LB) broth (Difco, Sparks, MD, United States) supplemented with 34 μg/ml chloramphenicol (MilliporeSigma, ON, Canada).

A mixture of *Salmonella* Enteritidis, *Salmonella* Heidelberg, and *Salmonella* Newport was used for the carcass trial. The three serovars were activated at 37°C in NB and sub-cultured by transferring 0.1 ml of 24 h suspension to 10 ml of fresh NB. They were then mixed together and centrifuged at 5000 ×*g* for 15 min at 20°C (Multifuge 1S-R, Heraeus, Osterode, Germany) and washed twice with sterile buffered peptone water (Hardy Diagnostics, Santa Maria, CA, United States). The final suspension in peptone water was used as test inoculum, adjusted to a viable count of about 8 log_10_ CFU/ml. *Salmonella* spp. were enumerated on XLT-4 agar (Hardy Diagnostics).

### Production and Quantification of Reuterin, Lactic Acid, and Microcin J25

A two-step fermentation process was used to produce reuterin as described previously (Vimont et al., 2019). *Lactobacillus reuteri* was cultured in 1 L of MRS medium supplemented with 20 mM glycerol and incubated overnight at 37°C. The cells were then harvested by centrifugation at 1500 ×*g* for 10 min at 20°C, washed with potassium phosphate buffer (0.1 M, pH 7.0), resuspended in 100 ml sterile aqueous solution of glycerol (300 mM). Reuterin was then collected after 2 h by centrifugation (10,000 × *g*, 10 min, and 4°C) and filtration through 0.2-μm pore size membrane filter. High-performance liquid chromatography (HPLC) was used to quantify the reuterin. The solution was analyzed by an HP1100 (Agilent Technologies, CA, United States) on a Coregel ION300 column (7.8 × 300 mm, Cobert Associates, Inc., Saint Louis, United States) with 10 mM H_2_SO_4_ as eluent at 40°C and a flow rate of 0.4 ml/min. Components were identified and quantified using a refractive index detector (Agilent Technologies). The compound was stored in solution at −20°C until use.

Microcin J25 was produced by *E. coli* MC4100 cultured in the minimal medium M63 following the method described and published by our laboratory (Hammami et al., 2015; Boubezari et al., 2018; Naimi et al., 2018; Ben Said et al., 2020). The bacteriocin was recovered from the supernatant of overnight culture using a Sep-Pak C18 35 cc vac cartridge (Waters, Milford, United States) at 4°C. Its concentration was calculated using an HPLC method previously described (Gomaa et al., 2017).

Lactic acid purchased from Laboratoire Mat Inc. (QC, Canada) was diluted in distilled water to achieve desired concentration before being used and sterilized by microfiltration (0.2 μm, MilliporeSigma).

The inhibitory activity of antimicrobial compound was verified visually using the agar well diffusion method (Naimi et al., 2018). About 25 ml of sterile medium containing 0.75% (w/v) agar was seeded with 1% (v/v) of an overnight culture of *Salmonella* Enteritidis and then poured into a sterile Petri dish. After solidification, wells were then cut and filled with 80 μl of the compound to be tested. Plate was incubated at 37°C for 18 h and the diameter of the inhibition zone was measured by a ruler.

### Determination of Minimum Inhibitory Concentration and Synergism

A microdilution method described previously (Ben Said et al., 2020) was used with minor modifications. Two-fold serial dilutions in NB starting from 125 μl of tested antimicrobial compounds were prepared in assay plates (96 wells, Becton Dickinson Labware, Franklin Lakes, NJ, United States). Each well received 50 μl of overnight *Salmonella* Enteritidis culture diluted 1,000-fold in fresh medium. The plates were incubated for 18 h at 37°C and the absorbance at 595 nm was measured every 20 min using an Infinite® F200 PRO photometer (Tecan US inc., Durham, NC). The minimum inhibitory concentration (MIC) was the lowest concentration that prevented visible bacterial growth.

Synergic activity was evaluated using the checkerboard assay (Garcia, 2010; Laishram et al., 2017). Wells containing 50 μl of each antimicrobial agent received 100 μl of overnight *Salmonella* culture diluted to 5 × 10^5^ CFU/ml. The plates were incubated at 35°C for 24 h under aerobic conditions (Hanchi et al., 2017). The fractional inhibitory concentration index (FICI) was calculated as follows:

$$\begin{array}{l} FICI=\frac{MIC\;of\;compound\;A\;in\;combination}{MIC\;of\;compound\;A\;alone}+\\ \;\;\;\;\;\;\;\;\;\;\;\;\;\;\;\;\;\;\frac{MIC\;of\;compound\;B\;in\;combination}{MIC\;of\;compound\;B\;alone} \end{array}$$

In the remainder of this paper, MIC_c_ (MIC_c_ A or MIC_c_ B) refers to each MIC of each compound when used in the synergetic combination (A + B). The interaction is synergic if FICI is ≤0.5, additive or indifferent if FICI is in 0.5–4 range, and antagonistic if FICI is ≥4 (Hanchi et al., 2017). The minimum and fractional inhibitory concentrations were calculated using duplicate medians obtained in three independent experiments.

### Measurement of *Salmonella* Growth Inhibition

Inhibition of *Salmonella* Enteritidis by reuterin, microcin J25, and lactic acid alone or in combination was evaluated in micro-assay plates. Bacteria were grown in LB, centrifuged (Multifuge 1S-R, Heraeus, Osterode, Germany) at 2500 ×*g* for 15 min at 4°C, washed, and re-suspended in 0.85% saline. Plate wells were received 10^5^ CFU/ml bacterial suspension, supplemented with antimicrobial agents, and incubated at 37°C. The concentration of each antimicrobial agent was MIC_c_ determined above. Growth was measured as absorbance at 595 nm (Tecan US inc., Durham, NC) every 45 min for 24 h based on three experimental repetitions. Viable counts were obtained in duplicate for 0, 3, 6, 12, and 24 h using the drop plate count method on LB agar (Difco, Sparks, United States).

### Bacterial Inactivation Time-Course Curves

LB containing reuterin + lactic acid or reuterin + microcin J25 at 5 or 10 times the MIC_c_ was inoculated with *Salmonella* at 10^5^ CFU/ml and held under ambient condition. Viable cells remaining after each exposure time were counted on LB agar. The exposure times were 10, 20, and 30 s; 1, 5, 10, 15, 20, and 30 min; and 3, 6, 12, 18, 20, 22, and 24 h.

### Effectiveness of the Inhibitors on Broiler Carcasses

A total of 24 commercial broiler carcasses obtained immediately after processing without antimicrobial treatment were assigned randomly to the four treatment groups. A Health Canada pathogen challenge test protocol (Health Canada, 2012) was modified slightly for the mixed *Salmonella* test inoculum used in this study. The carcasses were inoculated on the medial and lateral sides with 1 ml (five times 200 μl) of bacterial suspension and then placed under a biohazard hood for 20 min. The load of attached cells was about 10^5^ CFU per gram chicken carcass.

Carcasses were then treated by spray with 200 ml of the antimicrobial formulas including water, reuterin + lactic acid, reuterin + microcin J25, and peracetic acid. The procedure was repeated for a total of six carcasses per treatment (3 × 2 replicates; Lemonakis et al., 2017). The peracetic acid was a commercial product (CHINOOK, Sani-Marc Inc., Victoriaville, QC, Canada) prepared according to the manufacturer’s instructions. The natural inhibitors were applied at 10 times the MIC_c_ and the peracetic acid concentration was 0.1%. To reduce cross contamination, treated carcasses were placed in individual sterile poultry rinse bags (Nasco, Madison, United States) for holding at 4°C to mimic the refrigeration conditions used at industry level. They were tested for total aerobes and *Salmonella* after 24 h.

Each carcass was rinsed with 400 ml of buffered peptone water containing 0.1% sodium thiosulfate (Fisher Scientific, Fair Lawn, NJ) to neutralize residual antimicrobial compound (Lemonakis et al., 2017) and then shaken vigorously for 60 s in a sterile chicken-rinse bag. Rinsing solution was collected in a conical tube and a 10 μl aliquot was diluted serially in 90 μl of phosphate-buffered saline in a flat-bottom 96-well micro-assay plate. Diluted aliquot was plated on tryptic soy agar for counting aerobic bacteria and on XLT-4 agar for *Salmonella*. These plates were incubated at 37°C for 24 h, and colonies were counted manually. On XLT-4 agar, only colonies with a black center were counted as *Salmonella*.

### Statistical Analysis

Raw counts were log-transformed for statistical analysis. Reductions were determined as log_10_ (*N*_0_/*N*), where *N*_0_ was the average control count and *N* the experimental treatment count. Data were analyzed using SPSS software (version 22.0, IBM Corporation, Armonk, NY, United States) for ANOVA. Significant differences (*p* < 0.05) between treatment effects were declared on the basis of the Tukey’s test.

## Results

### Inhibitory Activity and Synergy of the Antimicrobial Agents

**Figure 1:**
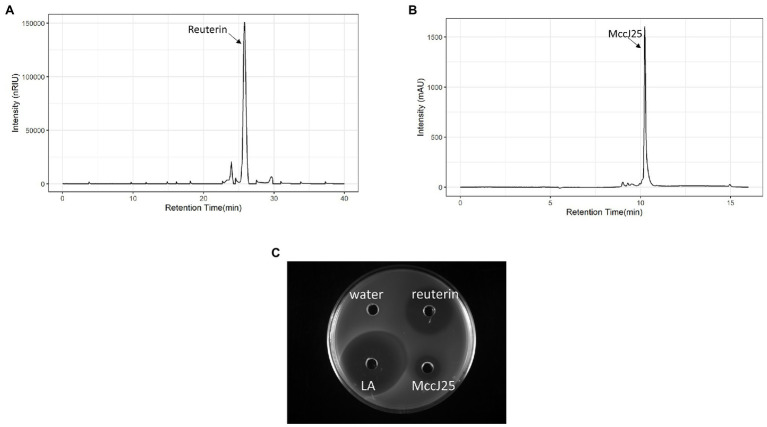
HPLC chromatograms of reuterin **(A)** and microcin J25 (MccJ25; **B)**; inhibition of *Salmonella* Enteritidis MNHN by reuterin, MccJ25, and lactic acid (LA) in nutrient agar **(C)**.

Reuterin and microcin J25 purity was evaluated by HPLC, and anti-*Salmonella* activity was confirmed by the agar diffusion test (Figure 1). The MIC values of the individual compounds and the two combinations against *Salmonella* Enteritidis are shown in Table 1. Reuterin alone had a MIC of 2 mM; lactic acid and microcin J25 inhibited *Salmonella* Enteritidis at 0.31% and 0.03 μM, respectively. When they were used in combinations, the MIC_c_ of the combinations were four times lower (0.5 mM, 0.078%, and 0.008 μM). According to the equation above, the combinations reuterin + lactic acid and reuterin + microcin J25 were both synergic (FICI = ¼ + ¼ = 0.5) at lower concentrations. Since no synergetic effect (FICI = 1) was observed for microcin J25 + lactic acid, this formula was not considered in this study.

**Table 1:** Inhibitory concentrations of the compounds against *Salmonella* Enteritidis.

	Antimicrobial combination	
	Reuterin (mM)-Lactic acid (v/v%)	Reuterin (mM)-Microcin J25 (μM)
MIC (A/B)	2/0.31	2/0.03
MIC_c_ (A + B)	0.5 + 0.078	0.5 + 0.008
FICI	0.5	0.5
Interaction type	Synergic	Synergic

MIC (A/B) = minimum inhibitory concentration of agent A or B alone; MIC_c_ (A + B) = each MIC of each compound when used in combination (A + B); FICI = fractional inhibitory concentration index.

### Inhibition of *Salmonella* Growth by the Synergic Pairs

**Figure 2:**
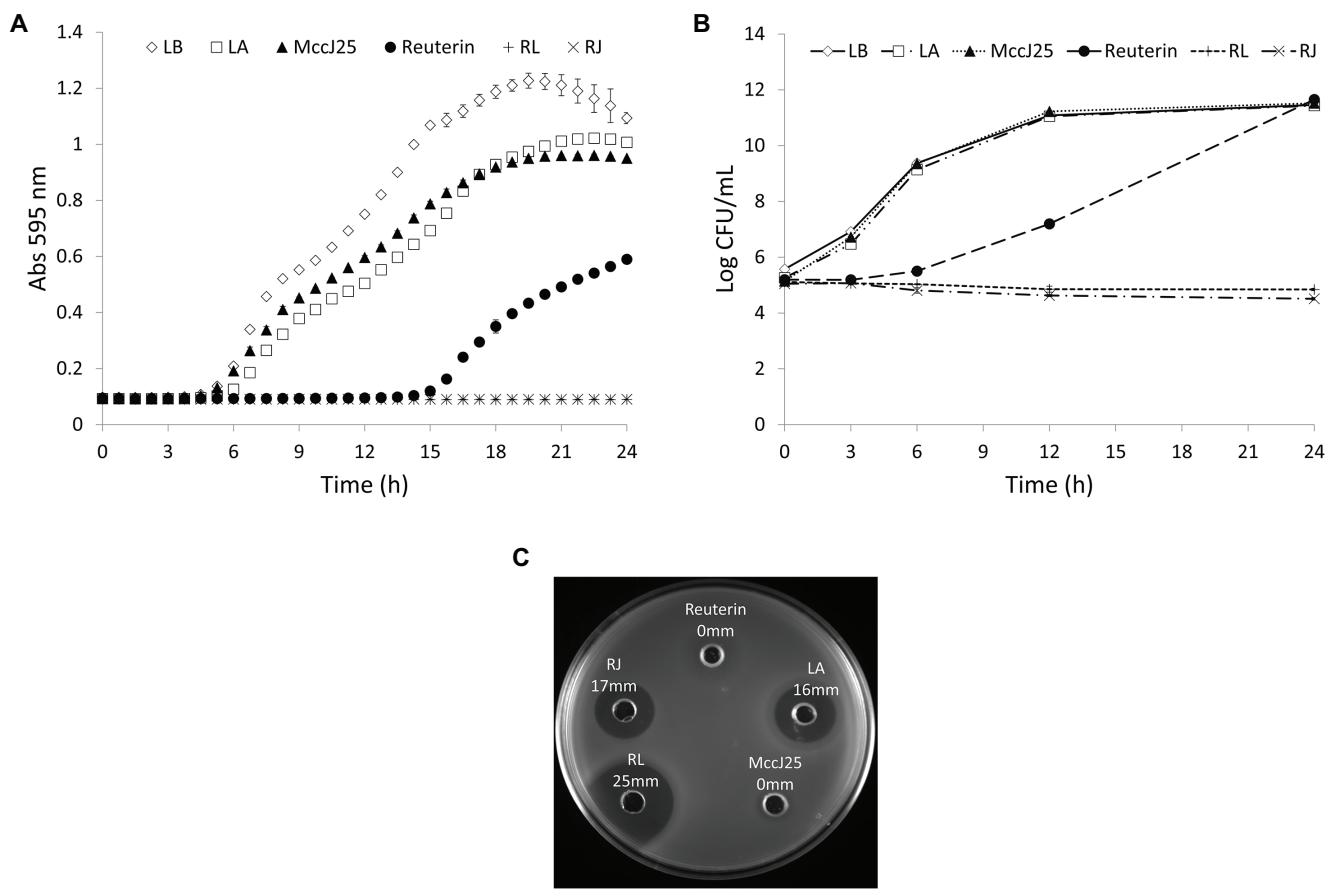
Inhibition of *Salmonella* Enteritidis MNHN growth at 37°C in LB broth by reuterin, lactic acid (LA), microcin J25 (MccJ25), reuterin + LA (RL), and reuterin + MccJ25 (RJ). **(A)** Optical density measurement; **(B)** viable counts; and **(C)** agar diffusion test. Error bars indicate standard deviation.

Figures 2A,B show the growth curves of *Salmonella* Enteritidis in LB broth containing reuterin, lactic acid, or microcin J25 alone or in combination at the MIC_c_ are listed in Table 1. Both combinations were entirely effective inhibitors of *Salmonella* growth for 24 h, whereas reuterin (0.5 mM) was the only effective single agent, stopping growth for 12 h. Neither lactic acid (0.078%) nor microcin J25 (0.008 μM) alone lowered viable counts, although optical density did appear to have been affected slightly.

In the absence of inhibitor, the viable count reached 11.5 log_10_ CFU/ml (Figure 2B). The combination of reuterin with lactic acid or microcin J25 inhibits the growth over time at these low concentrations. Although the bacteria were still present, slight drops of 0.27 and 0.54 log_10_ CFU/ml were observed for reuterin + lactic acid and reuterin + microcin J25 (Figure 2B). The agar diffusion result is shown in Figure 2C by using a high concentration to clearly distinguish the inhibition zone of the substance visually. There was no inhibition when reuterin or microcin J25 was used alone, but a 17-mm inhibition zone appeared when used in combination. And compared with lactic acid alone (16 mm), reuterin + lactic acid had a larger (25 mm) inhibition area.

### Concentration Dependency of Anti-*Salmonella* Bactericidal Activity

**Figure 3:**
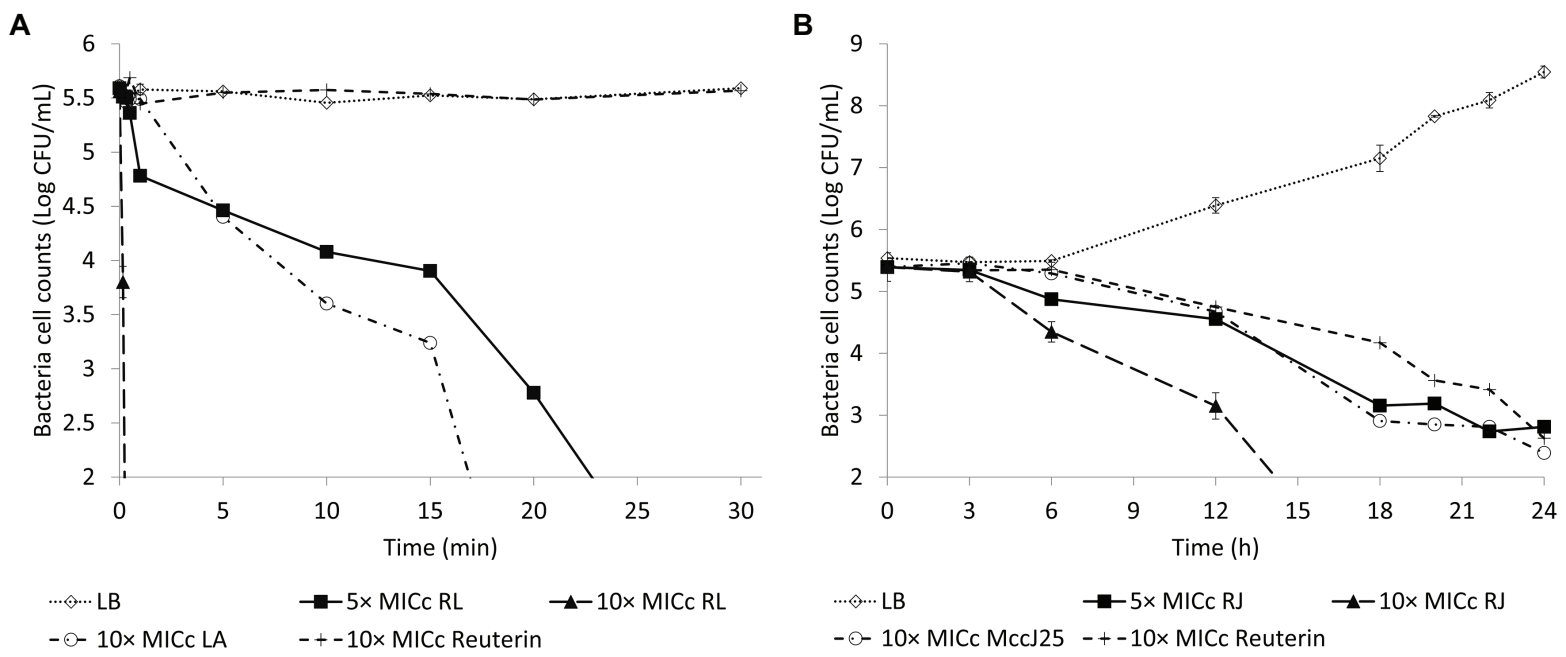
Survival of *Salmonella* Enteritidis MNHN at room temperature after exposure to (**A)** reuterin, lactic acid (LA), and reuterin + LA (RL); or (**B)** reuterin, microcin J25 (MccJ25), and reuterin + MccJ25 (RJ) at 5 or 10 times minimum inhibitory concentrations in combination (MICc). Bars indicate standard deviation.

The loss of viability of *Salmonella* Enteritidis in the presence of higher concentrations of the inhibitors is shown in Figure 3. Addition of five times the MIC_c_ of reuterin + lactic acid inhibited *Salmonella* within 30 min, and reuterin + microcin J25 (five times MIC_c_) induced 2.58 Log_10_ CFU/ml decrease of *Salmonella* counts within 24 h. At 10 times the MIC_c_ of the reuterin + lactic acid pair, counts dropped to zero in 20 s (Figure 3A). However, more than 12 h were needed to get a similar drop at 10 times the reuterin + microcin J25 MIC_c_ (Figure 3B).

**Figure 4:**
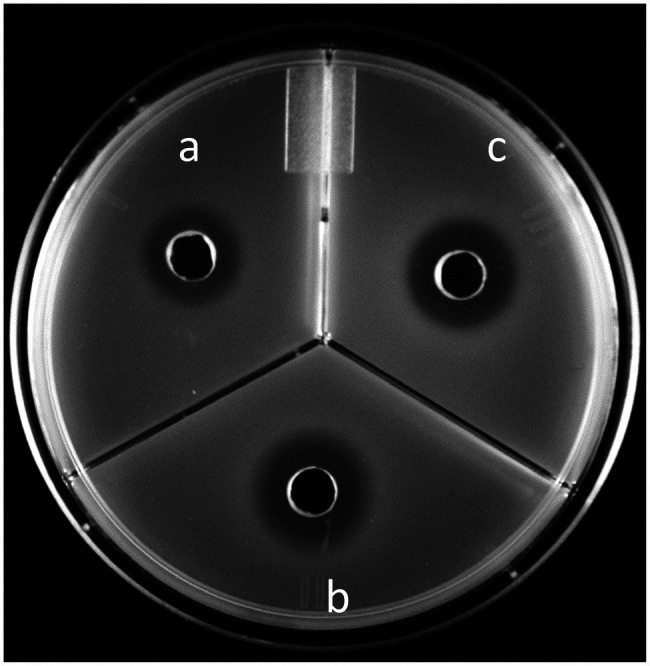
Microcin J25 agar diffusion test using **(A)** *Salmonella* Enteritidis MNHN, **(B)** *Salmonella* Heidelberg CMBL4-8, and **(C)** *Salmonella* Newport ATCC 6962

**Figure 5:**
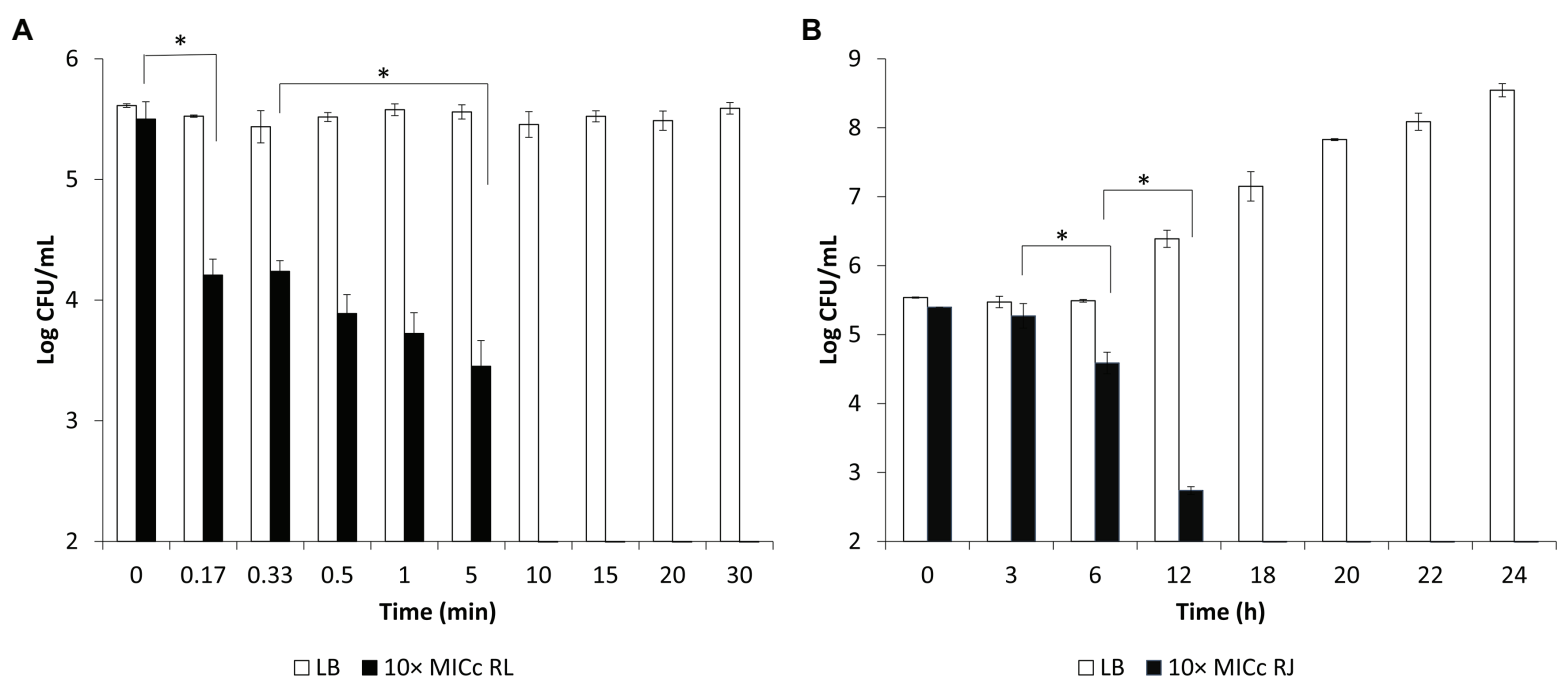
Counts of viable *Salmonella* mixture (*Salmonella* Enteritidis MNHN, *Salmonella* Heidelberg CMBL4-8, and *Salmonella* Newport ATCC 6962) after exposure in 10 times minimum inhibitory concentrations in combination (10 × MIC_c_) of reuterin + lactic acid (RL; **A)** and reuterin + microcin J25 (RJ; **B)**. LB broth as negative control. Bars indicate standard deviation. ^*^p < 0.05.

Validated by the agar diffusion inhibitory results of microcin J25 shown in Figure 4, *Salmonella* Enteritidis, *Salmonella* Heidelberg, and *Salmonella* Newport were selected to prepare a mixed inoculum, which was used for the following chicken carcass trial. The corresponding viable counts after treatment in broth culture at 10 times the MIC_c_ are shown in Figure 5. Reuterin + lactic acid significantly (*p* < 0.05) decreased the count of *Salmonella* immediately after treatment, and reuterin + microcin J25 had this significant effect after 6 h. These results confirmed that reuterin + lactic acid was the more potent of the two combinations, with an unequivocal bactericidal effect measurable within 10 min, while after 12 h for reuterin + microcin J25.

### Antibacterial Activity of the Inhibitors on Chicken Carcasses

**Figure 6:**
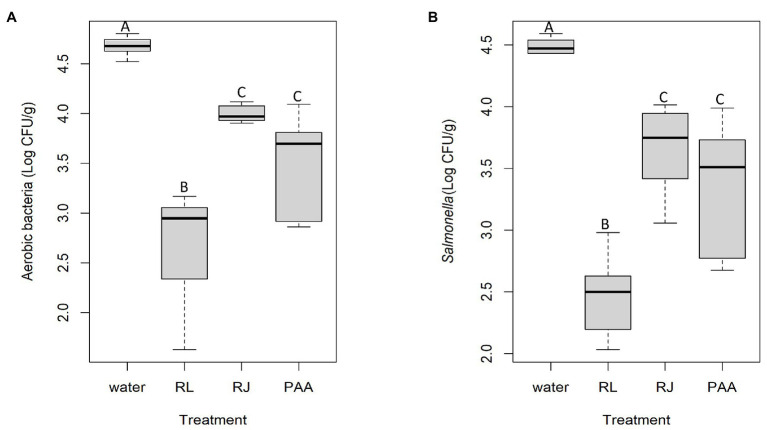
Total aerobic **(A)** and *Salmonella* **(B)** viable counts on chicken carcasses sprayed with antimicrobials (RL = reuterin + lactic acid, RJ = reuterin + microcin J25, PAA = peracetic acid; *n* = 6 for each treatment). Different letters indicate significant differences (*p* < 0.05).

Total aerobes and *Salmonella* on broiler carcasses treated with the inhibitors in comparison with peracetic acid are shown in Figure 6. The two antimicrobial combinations showed a significant effect (*p* < 0.05), and the reuterin + lactic acid appeared to be even better than the industrial product used as prescribed, reducing total aerobes (Figure 6A) by 1.99 Log_10_ CFU/g relative to the negative control treatment (water). The peracetic acid and reuterin + microcin J25 treatment achieved similar total aerobic bacterial counts ranging from 3.51 to 3.99 Log_10_ CFU/g on carcasses. Their difference of bacterial number reduction was not statistically significant (*p* > 0.05).

Much the same pattern was obtained for *Salmonella*, as shown in Figure 6B. The significant reduction was 2.02 Log_10_ CFU/g for reuterin + lactic acid relative to treatment with water, while reuterin + microcin J25 and peracetic acid had smaller and similar potencies with a reduction of 0.83 and 1.13 Log_10_ CFU/g (*p* < 0.05), respectively. The treatment of both reuterin + microcin J25 and peracetic acid did not differ significantly in recovered *Salmonella* (3.66 and 3.36 Log_10_ CFU/g of *Salmonella*).

## Discussion

The current shift in consumer preference for foods preserved using natural substances rather than chemicals is not likely to fade. Demand for more nutritious and safer food is expected to continue to grow, which will increase the need for more natural and affordable inhibitors of foodborne pathogens. Natural antimicrobial compounds offer several advantages over current treatments. One of these is that using them in combinations should lessen the likelihood of antimicrobial resistance developing among the targeted pathogens. The goal of this work was to study the effectiveness of two synergic combinations of natural antimicrobial compounds applicable by spraying to reduce the viability of *Salmonella* on chicken carcasses.

Decontamination of poultry carcasses involves primarily rinsing and chilling with water followed by spraying or dipping in solutions that may contain chlorine, organic acids, and in some cases bacteriocins. However, chlorine concentrations as high as 3,400 ppm have been found to fail to eliminate *Salmonella* Typhimurium on turkey even while causing unacceptable changes in the appearance of the meat (Teotia and Miller, 1975). Organic acids, especially lactic and acetic, have been widely used on chicken meat surfaces because of their availability and low cost, but their efficacy may depend on the type of surface and on the tenacity with which the bacteria attach (Burfoot et al., 2015). Bacteriocins, such as nisin, are active against *Clostridium* spp. and *Listeria* spp. (Özel et al., 2018) but do not inhibit Gram-negative bacteria attached to meat surfaces (He et al., 2016). Peracetic acid is an oxidizing agent that appears to denature proteins and enzymes and increase cell wall permeability by disrupting sulfhydryl and sulfur bonds (Rosario et al., 2021). However, it is corrosive, unstable, and an irritant in the upper respiratory tract even at low concentrations (Dittoe et al., 2019). The ideal treatment would be effective at low concentrations of agents having a broad antibacterial spectrum and low risk of being thwarted by the development of resistance strains.

In a previous study (Kuleasan and Cakmakci, 2002), reuterin alone was reported to inhibit the growth of *Listeria monocytogenes* on the surface of sausages. Lactic and acetic acids in tandem have been found to reduce *Salmonella* cocktail (*Salmonella* Enteritidis ATCC 13076, *Salmonella* Typhimurium ATCC 14028, *Salmonella* Typhimurium ATCC 13311, *Salmonella* Heidelberg ATCC 3347–1, and a wildtype *Salmonella*) on chicken parts (Ramirez-Hernandez et al., 2018). Microcin J25 has been shown to reduce *Salmonella* Typhimurium counts by approximate 3 Log_10_ CFU/ml in pork mincemeat extract (Yu et al., 2019). However, to our knowledge, no study has examined the efficacy of the combination of reuterin and bacteriocin. Inhibition of *E. coli* O157:H7 and *L. monocytogenes* by reuterin + lactic acid has been reported once, in the context of cooked pork surfaces (El-Ziney et al., 1999). Although 5% lactic acid added into 500 AU/ml of reuterin enhanced the antimicrobial activity by reduction of 1.88–2.9 Log_10_ CFU/cm^2^ for *E. coli* O157:H7 and 0.64–0.7 Log_10_ CFU/cm^2^ for *L. monocytogenes*, the concentration used for each compound was not determined by synergy test and considerably higher than the concentration of reuterin + lactic acid (5 mM + 0.78%) used in our work. In the present study, the use of a cocktail of three different *Salmonella* strains (*Salmonella* Enteritidis, *Salmonella* Heidelberg, and *Salmonella* Newport) is based on recommendation of Health Canada challenge protocol. According to this recommendation, using a mixture of at least 3–5 different strains allows to take into consideration variation in growth and survival characteristics among strains and therefore provide more consistent and representative results. These three strains are sensitive at different extents to microcin J25 as shown by agar diffusion assay. Microcin J25 is known for its inhibition activity against Gram-negative bacteria including *Salmonella*, while reuterin is known for its large spectrum of inhibition activity against both Gram-positive and Gram-negative bacteria. Combining these two antimicrobials will allow a stronger inhibition activity and large spectrum of inhibition. We have shown that combinations of reuterin with lactic acid or microcin J25 are synergic as inhibitors of *S. enterica* serovars. At 10 times the MIC_c_, both combinations reduced *Salmonella* counts to undetectable level within contact times that are applicable in the commercial context. Also, we can postulate that the mechanism of action of both combinations depends on the initial concentration. Indeed, at higher concentration (10 times MIC_c_), the inhibition activity is bactericidal since we did not see growth over the first 24 h. However, at concentrations lower than 10 times MIC_c_, the antimicrobial effect was bacteriostatic. These results were confirmed by *Salmonella* growth and concentration dependency assay.

The effectiveness of reuterin + lactic acid and reuterin + microcin J25 for reducing *Salmonella* counts on chicken carcasses suggests that these mixtures should be tested in poultry processing. Lactic acid is allowed at concentrations up to 5% to reduce *Salmonella* counts on animal carcasses (Lemonakis et al., 2017). Using sprayed 2% lactic acid, *Salmonella* Typhimurium has been reduced by 2 Log_10_ and total aerobes by 1.03 Log_10_ on chicken carcasses (Yang et al., 1998). In contrast, dipping in 5% lactic acid was found to reduce *Salmonella* by about 0.8–1.7 Log_10_ (Lemonakis et al., 2017). Microcin J25 used alone at concentrations of 8–16 μg/ml gave 3 Log_10_ reductions of *Salmonella* Typhimurium and *E. coli* O157:H7 (Yu et al., 2019). In our study, the concentration of lactic acid was less than 1%, which should have a smaller effect on sensory attributes, and both microcin J25 and reuterin were also used at lower concentrations in the combination. On the other hand, the antimicrobial combination at low concentration would consist ideally of compounds that work by fundamentally different mechanisms in order to lessen the likelihood of the development of resistance in bacterial species that pose serious threats to human health. In addition, in terms of cost, lower concentration and smaller volume are more cost-effective through the use of spray. Moreover, the raw materials used in the production of reuterin and microcin, such as glycerol, glucose, etc., are cheap and easy to obtain.

The method of application of the inhibitory product to processed poultry may have a measurable impact on the antimicrobial effect obtained. Immersion in acetic acid and in acidic electrolyzed oxidizing water was found to reduce *Salmonella* Typhimurium by respectively about 1.41 and 0.86 Log_10_, whereas spray-washing with the same solutions had no effect (Fabrizio et al., 2002). In the present study, spraying peracetic acid reduced the *Salmonella* load by about 1.13 Log_10_ CFU/g compared to the control. On post-chilled ground chicken, 0.1% peracetic acid brought a nearly 1.5 Log_10_ reduction (Chen et al., 2014). These observations suggest that chilling might increase the efficacy of subsequent treatments intended to reduce *Salmonella* counts on poultry (Lemonakis et al., 2017) and that the application method may play an important role and needs to be compatible with chilling. Other factors, such as antimicrobial concentration and contact time, also seem to be involved in the variability of the results reported. In the case of chicken drumsticks dipped for 15 min in solution containing 220 ppm peracetic acid, *Salmonella* Enteritidis counts were reduced by 0.36 Log_10_ CFU/g on day 0 (del Rio et al., 2007). In contrast, post-chill immersion for 20 s in peracetic acid at 400 ppm and 1,000 ppm could reduce *Salmonella* Typhimurium and *Campylobacter jejuni* loads on chicken carcasses by up to 2 Log_10_ CFU/ml (Nagel et al., 2013). In our study, the antimicrobial effects differed not only in terms of numerical reduction but also the time course. The diversity of the mechanisms of action might be one reason for the time variance. The reuterin aldehyde group reacts with primary amines and thiol groups, which are present on many small molecules and proteins (Schaefer et al., 2010), which could explain why reuterin has a broad-spectrum effect on microorganisms. Microcin J25 appears to have two intracellular targets, namely RNA polymerase and the respiratory chain. However, uptake of microcin J25 by the target strain requires the outer membrane receptor FhuA and the inner membrane proteins TonB, ExbD, ExbB, and SbmA (Ben Said et al., 2020), providing as many opportunities for resistance to develop as a result of structural mutations. In contrast, lactic acid is active in its undissociated form, which penetrates *via* the plasma membrane and reduces intracellular pH as well as disrupting the outer membrane of Gram-negative bacteria (Coroller et al., 2005). The antibacterial effect of the reuterin + lactic acid combination therefore occurs quickly (within 10 min), whereas reuterin in combination with microcin J25 needs somewhat more contact time in order to be effective against *Salmonella*.

Overall, this study shows that spraying a solution of natural inhibitors onto chicken carcasses results in significant but not vast reductions in viable counts of a pathogenic genus. By comparison, smaller than 1 Log_10_ reductions of total aerobic and *Salmonella* counts on broiler carcasses chilled for 45 min in aqueous ozone were observed decades ago (Sheldon and Brown, 1986) and modest reductions (0.53–0.69 Log_10_) were obtained using acidic solutions relatively recently (Ramirez-Hernandez et al., 2018). It has been shown that bacteria reside not only on exposed skin or muscle surfaces, but also within holes left in the skin by feather removal (Zhang et al., 2013) and that these shelter bacteria from the effects of subsequent antimicrobial treatments. That bacterial loads are smaller in skin-off than skin-on products is therefore hardly surprising (Ramirez-Hernandez et al., 2018). Complete decontamination of poultry carcasses by antimicrobial agents is likely to remain elusive for the foreseeable future.

## Conclusion

The results of the present study confirm that mixtures of reuterin with lactic acid or microcin J25 are synergic inhibitors of *Salmonella* and therefore warrant testing as candidates for improving the safety of poultry products. The combination of lactic acid with reuterin was more potent than peracetic acid. Synergic combinations of agents at low concentrations could contribute to slowing the development of resistance in pathogenic species and decrease the residual toxicity of food decontamination treatments. Thus, the two combinations, especially reuterin + lactic acid, appear to be suitable for application to broiler carcasses by spraying. Regulatory agencies should consider these results in developing the new strategic plan for *Salmonella* control in poultry meat products based on natural antimicrobials intended to improve occupational health and safety for poultry processing employees. Further study is needed to test these combinations against other pathogenic bacteria, such as *Campylobacter*, and to understand their impact on the shelf life of processed poultry and subsequent changes in the microbiome. Multiple antimicrobial combinations, sequential application, and different methods, such as dipping and immersion, may be needed in order to achieve the desired result.

## Data Availability Statement

The original contributions presented in the study are included in the article/supplementary material, further inquiries can be directed to the corresponding author.

## Ethics Statement

Ethical review and approval was not required for the animal study because the birds were raised in an off-campus commercial farm, and the current study was restricted to the microbiological evaluation of bird carcasses.

## Author Contributions

LZ, LBS, and IF designed the experiment. LZ performed the experiment and wrote the paper. LBS, MD, and IF edited the paper. All authors read and approved the final manuscript.

### Conflict of Interest

The authors declare that the research was conducted in the absence of any commercial or financial relationships that could be construed as a potential conflict of interest.
